# Developing a framework regarding a complex risk based methodology in the evaluation of hazards associated with medicinal products sourced via the internet

**DOI:** 10.1016/j.jsps.2020.10.018

**Published:** 2020-11-06

**Authors:** Róbert György Vida, Sára Merczel, Eszter Jáhn, András Fittler

**Affiliations:** a University of Pécs, Faculty of Pharmacy, Department of Pharmaceutics and Central Clinical Pharmacy, Honvéd Street 3, 7624 Pécs, Hungary; b Department of Pharmacy, Somogy County Kaposi Mór Teaching Hospital, Tallián Gyula Street 20-32, 7400 Kaposvár, Hungary

**Keywords:** Internet pharmacy, Risk assessment, Eye drops, Counterfeit medicine, Online medicines, Test purchase

## Abstract

Today, the increasing number of illicit internet pharmacies is a global phenomenon, however, the size of the online pharmaceutical market is still relatively unknown and the dubious quality of products is questionable and warrants investigation. Descriptive data from this black market channel are derived from studies analyzing the online availability of different medications procured over the internet and their methodology is quite heterogeneous. Our aim was to develop a comprehensive and specific risk assessment for selecting high patient safety risk medications from the online pharmaceutical market. A rapid tool was developed based upon the two quality and safety standard resolutions in pharmaceutical practice, published by the European Directorate for the Quality of Medicines, and was illustrated on eye drops. We developed five dimensions in support of the risk assessment including intrinsic, extrinsic and potential risks of counterfeiting. The five criteria were integrated in a comprehensively weighted risk-scoring format. The probability of procuring the product from the internet was also assessed based on the number of relevant links within the first twenty search engine results and the cost of the products. With the application of the tool a dorzolamide & timolol combination eye drop represented the highest overall patient safety risk score. In consideration of our literature review of the past 20 years, there is no current, standardized methodology to effectively identify pharmaceutical products associated with high patient safety risks. Notably, the fully comprehensive analysis of the internet pharmaceutical market and the test purchase of all online available medicines is unrealistic. Therefore, we developed a method to aid online surveillance researches and targeted international organizational led joint actions against the uncontrolled sale of falsified and substandard medications (e.g.: Operation Pangea).

## 1 Introduction

The expanding market of illicit internet pharmacies is a global public health threat with potential negative patient safety consequences in every aspect of our health care system. Although we primarily have indirect fragments of information regarding illegitimate vendors, it is highly likely illegal online pharmacies outnumber legitimate counterparts (Liang and Mackey, 2009, Gabay, 2015). Google searches in reference to the term, “online pharmacy” revealed more than half of the search results were linked to fraudulent sites (Abbasi et al., 2012). According to the survey of the National Association of Boards of Pharmacy (NABP), in 2017, 95.7% of 11,749 online pharmacies were noncompliant with the U.S. legislation and standards (The National Association of Boards of Pharmacy, 2018).

The majority of these “Not Recommended” sites were found to be dispensing prescription-only medicines without a valid prescription (The National Association of Boards of Pharmacy, 2018). In 2016, based on the available industry data (LegitScript), there were 30,000 to 35,000 illicit online pharmacies (Corazza et al., 2014, LegitScript, 2016), and the market was estimated to grow by twenty online vendors daily (LegitScript, 2016).

Also more and more consumers are turning to the digital market to purchase medicine, as it is believed 1–10% of the general population procured at least once a healthcare product using the internet (Orizio et al., 2009, Orizio et al., 2011, Fittler et al., 2018a).

Additionally, there are no exceptions, as nearly every therapeutic area is available over the Internet, from “lifestyle drugs” to life-saving medications (Fittler et al., 2018b, Mackey et al., 2015, Mackey and Nayyar, 2016).

The available public data and literature regarding the internet sales of pharmaceuticals mainly focus on the surface web and simulate the behavior of a potential consumer. Therefore, the data is typically derived from studies analyzing the online availability and quality of different medications or other healthcare products procured using the internet (Orizio et al., 2011, Norbutas, 2018, Vida et al., 2017, Vida et al., 2019).

The authors collected and reviewed publications on test purchases from the online pharmaceutical market in the last 20 years to find out whether these methods included any prior risk assessment to choose the model products for the evaluation of online accessibility or test purchase. These studies provide the highest level of evidence about the illicit online pharmacy market and the associated patient safety risks. We searched the PubMed for relevant literature with ‘internet pharmacy purchase’ and ’internet pharmacy quality’ key words in 2020. May. Further, review articles in this field from Orizio et al., 2011, Mackey and Nayyar, 2016 were included (Orizio et al., 2011, Mackey and Nayyar, 2016).

We included studies where the visit of the internet pharmacy and the attempt of purchase was carried out, regardless of actual purchase.

Online patient safety evaluation studies (n = 43) were categorized into four main groups, (1) pre-purchase vendor analysis for the estimation of patient safety issues, (2) actual test purchase with product quality analysis, (3) combined vendor, transport and product analysis, and (4) patient safety analysis using patient profiles/cases. In the latter category two studies from 1999 and 2005 used hypothetical patient profiles to evaluate whether there is a proper patient- and medication history checking when purchasing online sildenafil (1999 (Eysenbach, 1999) or hormonal contraceptives (2005, Memmel et al., 2006). In both cases the researchers could purchase the medications easily despite the several contraindications and interacting medications. It was also observed that there were no medical follow up after buying these medications, latter email or telephone contact by the vendors was for marketing purposes. Nine studies assessed only the quality of the purchased products (category 2) with various non-destructive methods (qualitative analysis without physical contact e.g.: near infra-red (NIR) and Raman spectroscopy) and destructive analytical techniques (qualitative and/on quantitative analysis where the integrity of medications was affected). The main methods were those recommended by the Pharmacopoeias and monographs (e.g.: HPLC for chemical analysis and methods to analyze formulations and dosage form integrity). 41.8% (18) of the studies were pre-purchase studies, as they did not order the actual product, they assessed if the product is available through the internet and what are the main characteristics of the online pharmacies (mainly illegal ones). We have identified a chronological development in the methods used, as assessments after the millennium focused on online prescribing and cyber doctors, price comparisons (mainly US and Canadian prices), shipping destinations, packaging and labeling. From 2005 more and more studies used the NABP VIPPS legitimacy verification (launched in 1999) (NABP) as an element in the assessment of the online vendors. From 2011 the CIPA and PharmacyChecker, while from 2013 the LegitScript was also used as a tool the differentiate between legal, illegal or unregulated internet pharmacies, and from 2014 the EU common logo also appeared in publications. We identified in the studies that the first domain analysis (WHOIS) was published in 2011, while the evaluation of other possible sources like social media (e.g.: Facebook, Twitter, Instagram) and global B2B trading platforms (e.g.: Alibaba) were incorporated first in 2013 by Mackey et al. (2015).

32.5% (14) of the test purchases used a combined method (14). Veronin et al. (2007) and the European Alliance for Access to Safe Medicines EAASM publications (EAASM, 2008) were the first ones to use these methods from 2006. The first comprehensive analyses was made by Gelatti et al. in 2011 (Gelatti et al., 2013), when they ordered fluoxetine pills from several online pharmacies and beside the website analysis, they completed a packaging, chemical and microbiological analyses of the ordered product. This and the Authors’ (Vida et al., 2017) analysis incorporated microbiological aspects of the internet market of the pharmaceuticals, however the latter did not carry out actual analysis.

When we look at the regional distribution of the studies, it can be seen that countries with more historical background of the mail order pharmacies were the primary locations, like the USA and Canada (18). The first European publication dates back in 2008 (altogether 11), while from 2009 we can see studies from Japan (5), while the first Australian study was published only in 2019. Regions from developing and transitional countries, and the Middle-East are represented with only one publication from 2018 (United Arab Emirates).

If we look at the search engines used in these studies, the leading role of the Google (17) is not a surprise, the second is Yahoo! (9) and the third most commonly used search engine was Bing (4) in this last twenty-year period.

Out of 31 only 18 test purchases (58.1%) included prior selection method. These included most commonly the following – partly overlapping - four criteria: (1) product with significant sale; (2) commonly used, recommended by guidelines; (3) there is high risk of ADR (e.g.: diazepam, fluoxetine); (4) most popular based on online searches.

Test purchases focused on oral dosage forms (20/25, 80%), only 4 (16%) included multiple dosage forms (e.g.: patch, inhalator) and 1 with just parenteral dosage form (somatropin). The most popular active pharmaceutical ingredients were the erectile dysfunction treatment sildenafil, the cholesterol lowering products simvastatin (5 purchases respectively), atorvastatin (4 purchases) and the antidepressant fluoxetine (3 purchases). Twenty two studies (51.2%) included multiple active pharmaceutical ingredients or products. Not surprisingly, the above list is in line with the global blockbuster drug sales in the year of the studies (Debnath et al., 2010). (*The summary of the published literature can be seen in a* Supplement 1.)

In 2017, our research group aimed to develop a more comprehensive method regarding the combination and categorization of the aforementioned characteristics associated with internet pharmacies aligned with chemical analysis (Vida et al., 2017). We have been using the perspective of the patient safety risk in our research, however we have not called it risk assessment and have not incorporated into a constructed tool. The selection of the active ingredient was based on the popularity, illegal use and parenteral dosage form, as we thought these characteristics are patient safety risk factors and facilitate the online sell and purchase of somatropin, while the products searched online were based on the national sales. The authors believe it is now timely to develop a patient safety risk assessment method in support of the selection of high-risk model products procured online, and to evaluate the online pharmaceutical market. Also, we believe these patient safety surveillance studies will provide an increased awareness to the potential risks associated with the digital market of pharmaceuticals. Moreover, this method can also be used as a preliminary analysis for the annual Operation Pangea (Mackey and Nayyar, 2016, Pangea, 2020) or for the further development of more sophisticated and emerging methodologies such as machine learning and web forensics (Takahashi et al., 2013, Nayyar et al., 2015, Nayyar et al., 2019, European Directorate for the Quality of Medicines (EDQM), 2016a, European Directorate for the Quality of Medicines (EDQM), 2016b, Mackey and Nayyar, 2017, Fittler et al., 2018b, Mackey et al., 2018, Li et al., 2019).

Our aim was to develop a complex method to select products associated with a high patient safety risk regarding the online pharmaceutical market. With the inclusion of risk factors originating from the product itself, consumer perspective, and potential of counterfeiting will result in improved surveillance and test procurement studies.

Thus, an original complex risk assessment methodology has been developed based on published literature and professional expertise to provide a recommended tool for research and authority test procurement. This general tool can be used for most medication classes, however adaptation to the specific properties to the given drug group is required. In order to illustrate the real world applicability of the developed risk assessment method, various ophthalmic medications have been evaluated, and the assessment of online availability regarding high-risk products have been completed.

## 2 Methods

### 2.1 Research instrument

Patient safety is comprised of several definitions throughout published literature. We define patient safety risk based on the broader perspective of medication errors, since patient safety risk is a risk which may cause or lead to inappropriate medication use and patient harm based on the characteristics of the product itself or the environment (beyond the traditional drug supply chain and control of health care system) while the medication is in the control of the patient or consumer. Based on these definitions, we assumed inappropriate medication use can lead to patient harm by increasing the likelihood of Adverse Drug Events (ADEs) (Hughes, 2008, World Health Organization (WHO), 2020, International Pharmaceutical Federation (FIP), 2020).

ADE is defined by the WHO as any untoward occurrence which may present itself during treatment associated with a pharmaceutical product which does not necessarily have a causal relationship to treatment. ADEs originate from the inappropriate use of the drug and its pharmacological property, or is associated with confounders, which occur during drug therapy but are not necessarily caused by the pharmacology of the drug itself (ACCP, 2015).

To assess the origin of an ADE, one must separate the different characteristics of a drug product and the association of ADEs with the specific attributions of the drug molecule itself and the possible contribution regarding the formulation. Reportedly, there are cases with ADEs reported after the use of modified release oral dosage forms, in particular, the ones with a delayed effect (e.g.: osmotic minipump tablets), or injections and inhalation products with different preservatives (e.g.: benzalkonium chloride or benzyl alcohol). Furthermore, when the bioavailability is compromised due to inappropriate use, as in the case of intramuscular injections, the risk of ADE is also increased due to the variability of serum concentrations. Topical formulations bear the risk of irritation and the possible ADEs originated from the systemic absorption of the active and non-active API (Uchegbu and Florence, 1996).

Since there is no specific patient safety risk assessment methodology for medications sold on the internet, a new tool was developed based upon the two quality and safety standard resolutions for pharmacy preparations in pharmaceutical practice, published by the European Directorate for the Quality of Medicines in 2016 (European Directorate for the Quality of Medicines (EDQM), 2016a, European Directorate for the Quality of Medicines (EDQM), 2016b). The idea was to incorporate the product related risks such as microbiological contamination, dosage form, pharmacological effect, therapeutic window and safety profile of the preparation of parenteral and other compounded dosage forms into a checklist that can help to identify which patient safety risks are relevant in case of the internet purchase of a drug product. The tool includes patient safety risks originated from the product itself (intrinsic risks), the potential for internet purchase (extrinsic risks) and the risks of counterfeiting based on the current WHO definition (WHO, 2017). The extrinsic risks were identified based on the authors previous experiences with test purchases and pharmacist expertise (Vida et al., 2017, Fittler et al., 2018a, Fittler et al., 2018b). Based on published literature, the two main potential motives for consumers when considering procuring pharmaceuticals using the internet market are low cost and the unavailability of medication in the legal supply chain (Mackey and Liang, 2012, Ashames et al., 2019, Bowman et al., 2019). Inexplicably, anonymity of the internet is perceived as a benefit as consumers may turn to illegal internet pharmacies aiming to procure purchase substances intended for recreational use, or abuse (Jena et al., 2011, Corazza et al., 2014, Kalyanam et al., 2017).

Beside the severity of patient safety risks, the probability of online procurement is also a part of the tool. In consideration of the evaluation of the online market and the probability of the patient safety risk, a partial (second type of research mentioned in the introduction) test purchase method was used. In order to the simulate the consumers, we used a Google engine with the search terms “buy” and “API International Nonproprietary Names (INN) name” in English. The first fifty search engine results were examined in May 2018, and sites offering eye drops with the defined API directly to patients (internet pharmacies) were included in our study. The number of relevant search results and product costs were documented. During our search, the authors were not signed into any account and the browser was set to standard security settings. Social media sites, blogs and forums were also included.

### 2.2 Data

Product specific information were extracted from the Summary of Product Characteristics (SmPC) and Patient Information Leaflet (PIL). As previous studies showed, medicines and other health products purchased over the internet typically arrive without a PIL or misleading labelling. Without detailed and appropriate counseling, the proper use of ocular dosage forms cannot be effectively guaranteed, increasing the risk of adverse drug events. Furthermore, if and when there is no product information on the website or disclosed product information, the consumer may not be cognizant of the recommended storage likely compromising product quality and safety. However, it is also not known whether the product was stored and handled properly during the transportation or before it. The transportation, storage temperature and circumstances (humidity, light, mechanical shock) can affect the physical, chemical and microbiological stability of the product. The latter characteristics are affected by the preservative content and whether the product is single-dose or multi-dose (Lagan et al., 2014, Mackey and Nayyar, 2016, Piñero-López et al., 2016, Tsegaw et al., 2017, Vida et al., 2017, Agarkhed et al., 2018, Sengupta et al., 2018).

### 2.3 Analysis

In order to demonstrate the applicability of our method we have selected a therapeutic drug category that we thought to have higher patient safety risk when purchased outside the closed drug supply chain. Their compromised quality, inappropriate use or misuse may lead to local or systemic health consequences (Kadri et al., 2010, Morales et al., 2016, Vaajanen and Vapaatalo, 2017, Gao et al., 2018).

Ten commonly used eye drops available in community pharmacies throughout Hungary were selected as model products to illustrate the tool. Various medications, including prescription-only and over-the-counter products and eye drops with supply disruptions were included in our study sample. The characteristics of the selected products were also diverse, including eye drops used in the treatment of glaucoma, allergy, infection, or used for diagnostic procedures (mydriasis). The selected products are highlighted in Table 1.

**Table 1:** The brand name and active ingredients of the ten selected eye drops.

Product brand name (Hungarian)	Active pharmaceutical ingredient
BETOPTIC 5 mg/ml (eye drop)	Betaxolol
AZOPT 10 mg/ml (suspension eye drop)	Brinzolamide
CILOXAN 3 mg/ml (eye and ear drop)	Ciprofloxacin
ALLEOPTI 20 mg/ml (eye drop)	Sodium cromoglicate
HUMAPENT 5 mg/ml (eye drop)	Cyclopentolate
SPERSALLERG 0.5 mg/ml + 0.4 mg/ml (eye drop)	Antazoline & tetryzoline
VISINE CLASSIC 0.5 mg/ml (eye drop)	Tetryzoline
COSOPT UNO 20 mg/ml + 5 mg/ml (single dose eye drop)	Dorzolamide & timolol
XALACOM 0.05 mg/ml + 5 mg/ml (eye drop)	Latanoprost & timolol
TRAVATAN 40 µg/ml (eye drop)	Travoprost

### 2.4 Validity

The content validity of our tool was checked by the four authors, as the dimensions, sub-dimensions and the scoring system were tested separately by each author and the final scoring system based on a consensus. The applicability of the tool was tested with the 10 eye drops *(see in* Supplement 2*)*.

## 3 Results

### 3.1 The dimensions of patient safety risk assessment of medicinal products

The proposed dimensions of the framework are suitable for all drug classes, however to improve the specificity of the risk assessment, customized sub-dimensions are required in accordance with the evaluated therapeutic categories or selected dosage forms. In nearly each dimension and sub dimension (except the complexity of application, where we used a 3 point scale) of the patient safety risk assessment, we selected Yes or No questions equaling 1 and 0 points in the scoring system. The total point for each patient safety risk dimension is based upon the number of sub dimensions (see Table 2 and Supplement 2).

1. **General pharmaceutical risk**

**Table 2:** Dimensions and sub-dimensions framework for assessing patient safety risks associated with medications procured over the internet tailored to ophthalmic preparations.

Dimensions of risk assessment		Sub-dimensions focusing on the risk factors specific to the evaluated drug class
INTRINSIC RISKS	1. General pharmaceutical risk	1.1. Dosage form
		1.2. Complexity of application
	2. Therapeutic risk	2.1. Mode of action
		2.2. Systemic absorption
		2.3. Altered absorption (Indication including damaged eye)
		2.4. Narrow therapeutic index (NTI)
		2.5. Special patient group (Pediatric indication)
	3. Risk of microbiological contamination	3.1. Single-dose vs. Multi-dose or Antimicrobial filter
		3.2. Preservative content
		3.3. API is an antibiotic

EXTRINSIC RISKS	4. Augmented demand for online purchase	4.1 Limited access (drug shortage, prescription requirement)
		4.2. Misuse potential (off-label indications, illegal use)

RISK OF COUNTERFEITING	5. Unregistered/unlicensed: Medical products which have not undergone evaluation and/or approval by the National or Regional Regulatory Authority (NRRA) for the market in which they are marketed/distributed or used, subject to permitted conditions under national or regional regulation and legislation	5.1. Based on preliminary evaluation whether the drug product is unregistered, investigational, or withdrawn.
	Falsified^1^: Medical products deliberately/fraudulently misrepresent their identity, composition or source.	Determined only by physical examination or by the verification of the serialized product.
	Substandard^1^: Also referred to as, “out of specification”, these are authorized medical products which fail to meet either their quality standards or specifications, or both.	Only complete analytics can assess safety risk.

1 *Risk of falsification and substandard quality cannot be integrated in the pre-purchase assessment, as such properties are undeterminable without physical and analytical examination. Accordingly, complete counterfeit risk assessment can be performed following actual purchase and delivery of products.*

The general pharmaceutical risk dimension describes the ADE risk originated from the dosage form, administration route and application or administration techniques of the medical products. These risks are augmented when medications are procured and used without supervision of a health care specialist (American Society of Health System Pharmacists, 1993, Dedefo et al., 2016).

The Institute for Safe Medication Practices (ISMP) maintains a list of high-alert medications in acute care setting, and medications liable to cause significant patient harm if not used properly. These mainly include parenteral and narrow therapeutic index (NTI) drugs (ISMP, 2018). Although eye drops are not on the previously mentioned list, the topical application of ocular drugs may cause adverse ocular or systemic side effects. It should be noted that not just the improper application, but the proper use of eye drops may cause systemic absorption. Systemic absorption can be a result of the high concentration of API and the different absorption mechanism through the cornea, conjunctiva and nasal mucosa (Blix et al., 2010, Farkouh et al., 2016).

Patients afflicted with tissue injuries, compromised metabolic capacity or immature blood–brain barrier function (e.g.: children or elderly) may experience systemic side effects after the application of topical formulas. In this section the medicinal product earns 1 point if it is a parenteral (e.g.: injection or topical) or modified release dosage form and 0 point if it is a conventional oral dosage form (Batchelor and Marriott, 2015).

The complexity of application was assessed on a three point scale based on the number of instructions for appropriate application in the SmPC and PIL. In case of the evaluated ophthalmic medications, the following categories were determined: >10 instructions – 3 points, 5–10 instructions – 2 points, <5 instructions – 1 point. Comprehensively, a product can be allocated a maximum 4 points regarding this dimension.

2. **Therapeutic risk**

Therapeutic risk dimension describes the ADE risk originated from the pharmacological property of the API. It consist of the assessment of the therapeutic window, indication in special patient groups. Drugs with narrow therapeutic index (NTI) are drugs with small differences between therapeutic and toxic doses. These products are more likely to cause ADEs than non-NTI-drugs (Iyer et al., 2018).

Further risk factor analysis showed in addition to the patient’s age and gender, health service-related (barrier to service), genetic factors (e.g.: CYP enzymes), disease related (e.g.: infectious diseases) and medication related factors, such as the inappropriate use of the medication or intravenous drug administration are more likely manifest in ADEs. Special patient groups such as the elderly, pregnant women and pediatric patients are also more likely to develop ADEs (Alomar, 2014, Zhou and Rupa, 2018).

To measure the therapeutic risk, we identified five main characteristics based on the SmPC reflecting the biopharmaceutical and pharmacological properties of the API and the preparation (Raynor et al., 2014). Ophthalmic preparations can be applied to have a local effects on the surface of the eye, as in case of artificial tears, or to reach systemic effect in the eye (e.g.: drugs to treat glaucoma). Although, the aim of the treatment is the eye itself, there is ample evidence when locally applied ophthalmic preparations had unwanted systemic effects. For example, vasoconstrictor eye drops can elevate blood pressure (cyclopentolate) or beta-blockers may cause bradycardia and bronchoconstriction (timolol) (Farkouh et al., 2016, Vaajanen and Vapaatalo, 2017). Since these products have relatively poor penetration, the products may contain high concentration of an active pharmaceutical ingredient (Labetoulle et al., 2005, Farkouh et al., 2016).

Specifically, information regarding *(1) mode of action* can be retrieved from the SmPC. When the product is intended for local effects, there is a lower chance for systemic absorption and adverse drug event (0 points), compared with, when there is a case of systemic ophthalmic effect, that has a higher risk of systemic absorption and occurrence of adverse drug event, therefore the product reaches 1 point. When the SmPC contains data or a warning regarding the systemic side effect or adverse drug reaction based on *(2) systematic absorption,* similarly to the previous criteria, the product gets 1 point, if there is no systemic ADRs, the point allocated is 0 point (Davies, 2000, Labetoulle et al., 2005, Farkouh et al., 2016, Vaajanen and Vapaatalo, 2017).

We used the SmPC to identify possible local adverse drug reactions including criteria based on the frequency categories recommended by the Council for International Organizations of Medical Sciences (CIOMS): very common (≥1/10); common (≥1/100 to <1/10); uncommon (≥1/1000 to <1/100); rare (≥1/10,000 to <1/1000); very rare (<1/10,000); Frequency not known (cannot be estimated from the available data) (Neubert et al., 2013, Sterling and Irwin, 2015, Agrahari et al., 2016) and the severity (minor, moderate, severe). In the case of at least one very common, or common or severe local ADR, the product gets 1 point (Petrova et al., 2017). When there is an inflammation in the eye, the penetration can also be heightened via the increased blood flow and lymphatic channels (Hornof et al., 2005, Farkouh et al., 2016). If the product is used to treat damaged or on a recently operated eye, the risk of systemic absorption and adverse drug reaction was high (1 point). If the *(3) indications included inflammatory ophthalmic disorders* there is also a greater chance of systemic absorption and adverse drug reaction (1 point) (Sterling and Irwin, 2015, Agrahari et al., 2016). In this section we also assess whether the products contain *(4) NTI active pharmaceutical ingredient* (1 point in the case of Yes). From the European Directorate for the Quality of Medicines resolution aiming the pharmacy preparation, we used the criteria “type of preparation” and considered the product more likely to cause systemic adverse drug reaction if the product had *(5) pediatric indication* (<6 years old) in the SmPC (1 point) (European Directorate for the Quality of Medicines (EDQM), 2016a, Farkouh et al., 2016). Comprehensively, a product can achieve a maximum 5 points (5 Yes or No questions) in this dimension.

3. **Risk originated from the likelihood of microbiological contamination**

Microbiological safety is one of the greatest concerns of the two EDQM resolutions. Therefore, we included a combined microbiological contamination risk originating from both the dosage form, the technology of the medication and the API’s pharmacological property (Kaushik et al., 2011, Teuchner et al., 2015, Tsegaw et al., 2017).

In case of counterfeit and illegally procured medications, serious patient safety risks may arise from the microbiological contaminations. This is a significant public health risk regarding individual infections and the global spread of microorganisms from developing countries to developed ones and vice versa which originates from the poor hygienic conditions during manufacturing, distribution, especially in case of sterile dosage forms (e.g.: eye drops and evidently parenteral medications). Studies show in the case of falsified and internet purchased medicines, there is a greater risk of microbiological contamination, and not just for tablets and capsules, but also for parenteral dosage forms (Mugoyela and Mwambete, 2010, Kaushik et al., 2011, Pullirsch et al., 2014, Teuchner et al., 2015). A retrospective study performed in Shanghai, China, investigated patients undergoing intravitreal injection in 2010 and found endotoxin as the cause of intraocular inflammation following the injection of a counterfeit bevacizumab (Wang et al., 2013).

Additionally, microbiological contamination is of major concern during the preparation and use of ophthalmic preparations, as it may lead to bacterial and fungal ophthalmic infections. Notably, it is not merely the microbiological stability that plays an important role and should be assessed, but the chemical and physical characteristics which are susceptible to environmental changes, the different formulations (e.g. single-dose, multi-dose) or the preservative content (e.g. preservative free) of the product also should be taken into consideration, especially when the only difference between two eye drops is the single-dose or multi-dose form (Brudieu et al., 1999, Nentwich et al., 2007, Tsegaw et al., 2017).

Further evaluated parameters in this dimension include an active pharmaceutical ingredient with antibiotic effect or the use of a special antimicrobial filter (Baudouin et al., 2010, Saisyo et al., 2016, Saisyo et al., 2017, Kyei et al., 2019).

When a product is single-dose, the risk of microbiological contamination is low, accordingly the product is allocated 0 point, while in case of multi-dose products it is 1 point. If the product has a special antimicrobial filter, the risk of contamination is low (0 point), and if there is no filter, the product gets 1 point. Similarly, if it contains a preservative, it is 0 point, and if there is none, the products is 1 point. When the active pharmaceutical ingredient is an antibiotic, the risk of microbiological contamination is lower (0 point), and when it is not, it is more likely to happen (1 point). Consequently, products can acquire a maximum 3 points in this dimension, as the authors think that the 3 methods to prevent microbiological contaminations (3.1. Single-dose vs. Multi-dose or Antimicrobial filter; 3.2. Preservative content; 3.3. API is an antibiotic) are equally effective and therefore no differences can be made in score system.

4. **Risk originated from the limited access to the product**

To assess the risk of an internet procurement of a medication, we must first know whether there is an increased demand for the active ingredient or product beyond the traditional supply chain. In case of prescription-only medications (or products only available in hospital care), products with several off-label or illegitimate indications, drugs in shortage, patients/consumers or even health professionals may turn to the internet market to overcome such restrictive barriers (Liang and Mackey, 2012a, Liang and Mackey, 2012b).

For the assessment of limited access and drug shortages, we developed a complex method where we evaluate whether if it is an official shortage or it affects essential medicines and how it can be solved such as with generic substitution, compounding or other alternative therapy. We also considered the consequences of a shortage, for example when the alternative therapy is less safe or there is an increased risk of Medication errors (MEs) due to change of the originally prescribed or ordered product (Liang and Mackey, 2012b, European Association of Hospital Pharmacists (EAHP), 2018, Fox and McLaughlin, 2018, Roth et al., 2018). However the complex evaluation is not in balance with the weight in this patient safety risk assessment. To exclude the dominance of this element, we simplified the points that can be earned in this section (1 point if it was in shortage in the evaluated period, and 0 point if not).

As it was previously mentioned, once a product has several indications, potential off-label or illegal uses, there is a greater chance it will be available on the Internet. To identify off-label use, we evaluated the authorized list from the Hungarian National Institute of Pharmacy and Nutrition. The addiction and abuse potential was assessed based on the information presented in the SmPC, while a literature search was used to determine if the API of the product is used illegally. In this category, the product is allocated 0 or 1 point (Mitchell and Dunnavan, 1998, St George et al., 2004, László, 2007, Mackey and Nayyar, 2016, Aronson and Ferner, 2017, Vida et al., 2017, Pangea, 2020, Institute, 2020a).

5. **The risk of counterfeiting**

The risk of counterfeit medicine dimension is based on the interpretation of WHO 2017 counterfeit medicine definition. The definition differentiate between three main categories including unregistered/unlicensed medicines, falsified medicines and substandard medicines in the broader definition of counterfeit pharmaceuticals. From these we selected the unregistered/unlicensed medicines to be include in the risk assessment, since it can be evaluated without the actual procurement of the product. With preliminary evaluation using the national drug authority databases (NIPN in Hungary), the researcher can determine whether the product offered online is an investigational drug product, a product not registered in the defined country (in our case, Hungary), or if it was withdrawn from the market (NIPN, 2020b).

If a drug product is identified as potentially counterfeit, the patient safety score is automatically 15 points and reaches the highest patient safety risk. The falsified and substandard dimensions were excluded from our risk assessment, since they can only be determined following procurement and receipt of the product, with physical examination or electronic verification (Falsified Medicine Directive or Drug Supply Chain Security Act), accompanied by a complete quality test with analytics and microbiological testing (House, 2014, Smith et al., 2014).

The detailed score system of the comprehensive patient safety risk assessment specific to eye drops and the result of the estimation of consequence/severity is shown in Supplement 2.

### 3.2 The measure of patient safety risk for the selected eye drops

The general pharmaceutical risk score varied between 2 and 4 out of maximum 4 points in case of the selected products. Ciprofloxacin eye drop according to the SmPC posed a risk of systemic absorption as the indications included damaged or operated eye (treating inflammatory eye diseases and corneal ulcer). The risk originated from the complexity of application was allocated the highest with 3 points in case of dorzolamide & timolol (S01ED51) eye drops as their SmPC contained more than 10 instructions.

In case of betaxolol (S01ED02), ciprofloxacin (S03AA07), latanoprost & timolol (S01ED51), and dorzolamide & timolol (S01ED51) the eye drops got the maximum 1 point, as the supervised use of these products can also cause systemic ADRs. It should be noted that three products contain beta 2-adrenergic receptor antagonists, and as previously highlighted, local beta-2-adrenergic antagonists are proven to cause bronchoconstriction in asthma patients.

In case of risk from the likelihood of microbiological contamination, only the ciprofloxacin got 1 point out of the 3, as the active pharmaceutical ingredient is an antibiotic, therefore increasing microbiological stability. All the products were multi-dose and contained a preservative (primarily benzalkonium chloride and/or BKC), and did not have any special antimicrobial filter. When we evaluated the products’ limited access, only the cyclopentolate (S01FA04) and dorzolamide & timolol (S01ED51) preparations were in shortage for more than two weeks throughout Hungary in May 2018. The latter combination also appeared on the WHO Essential Medicine List. Both products were allocated 1 point. We did not find any evidence regarding abuse potential nor misuse for any of the evaluated medications. When evaluating the online market during the partial test purchase method, the authors did not identify any unregistered/unlicensed medicines. Out of the maximum 15 points, the dorzolamide & timolol (S01ED51) product was allocated the highest score at 10 points, followed by cyclopentolate (S01FA04) with 9 points. The weighted patient safety risk was also calculated (the total patient safety risk score was divided by 10 points) and three distinctive categories were established with low (0.00–0.25), medium (0.26–0.74) and high (0.75–1.00) risk categories. None of the eye drops scored below 0.50. The detailed calculation of the patient safety risk scores for internet procurement of the selected eye drops can be seen in Supplement 2.

### 3.3 The probability of the online purchase of eye drops

Following the identification of the severity/consequence of the patient safety risks associated with the internet procurement of various medicines, the probability of product availability must also be assessed. We hypothesized that the number of vendors offering a given product online reflects its internet market share. The number of relevant links (links leading customers to websites offering medication for sale) within the first 50 Google search engine results were documented for each medication. The product availability point was calculated as a proportion compared to the highest number of relevant links of a given product (20) in our study, and three categories were established: high accessibility products with 15 or more links (1), medium accessibility products with number of links between 5 and 14 (0.5), and products with low accessibility with less than 5 links (0.25). Interestingly, it is not just supply, but demand also influences the online pharmaceutical market, thus the perspective of the consumers was also included in our methodology. We assumed, consumers are more likely to procure from those vendors offering their products at a lower or substantially reduced price. Consequently, we documented the total price (including shipping and handling fees) for each relevant retailer and categorized the products based on their online internet market affordability (1 = low price range; 0.5 = medium price range; 0.25 = high price range). The product was allocated 1 point if the price was less than $25, and 0.5 if the price was between $25 and $50, and 0 if it’s cost was more than $50. Cost categories were determined based on 5% and 10% of the Hungarian minimal wage in 2018, and in accordance with the national copayment database. Costs charged to patients in the legal national supply chain with a reimbursement were lower than $25 in case of all the products. The total weighted probability of online procurement was calculated by multiplying the availability and affordability scores, resulting in three main categories (0.0–0.25 = low; 0.26–0.74 = medium; 0.75–1.00 = high).

The detailed calculation regarding the probability scores for internet procurement of the selected eye drops is exhibited in Table 3.

**Table 3:** The calculation of the probability of the online procurement of eye drops.

Active pharmaceutical ingredients	Number of links in the first 50 Google search results and the product availability point (divided by 20)		Availability category 1 = high accessibility 0.5 = medium accessibility 0.25 = low accessibility	Average price of products available and online internet market affordability point		Affordability category 1 = low price range 0.5 = medium price range 0.25 = high price range	Total weighted probability of online purchase	Probability category 0–0.25 = low 0.25–0.75 = medium 0.75–1.0 = high
Betaxolol	15	0.75	1	>$50	0	0.25	0.25	Low
Brinzolamide	15	0.75	1	$25–50	0.5	0.5	0.5	Medium
Ciprofloxacin	4	0.2	0.25	<$25	1	1	0.25	Low
Sodium cromoglicate	11	0.55	0.5	<$25	1	1	0.5	Medium
Cyclopentolate	4	0.2	0.25	>$50	0	0.25	0.0625	Low
Tetryzoline & antazoline	4	0.2	0.25	$25–50	0.5	0.5	0.125	Low
Tetryzoline	4	0.2	0.25	$25–50	0.5	0.5	0.125	Low
Timolol & dorzolamide	20	1	1	<$25	1	1	1	High
Timolol & latanoprost	3	0.15	0.25	<$25	1	1	0.25	Low
Travoprost	7	0.35	0.5	$25–50	0.5	0.5	0.25	Low

The online availability of eye drops in general, is relatively low, however medications treating glaucoma containing betaxolol or brinzolamide are more commonly offered for sale, while timolol & dorzolamide products online availability was the highest amongst all eye drops included in our study. Another popular product on the internet is the cromoglicate antiallergic eye drop, with 11 links. The cost range of the evaluated eye drops are generally higher than the reimbursed community pharmacy prices throughout Hungary (on average, twofold), price categories (likelihood of procuring online) were defined, aiming to simulate national (Hungarian) customer decisions.

**Fig. 1:**
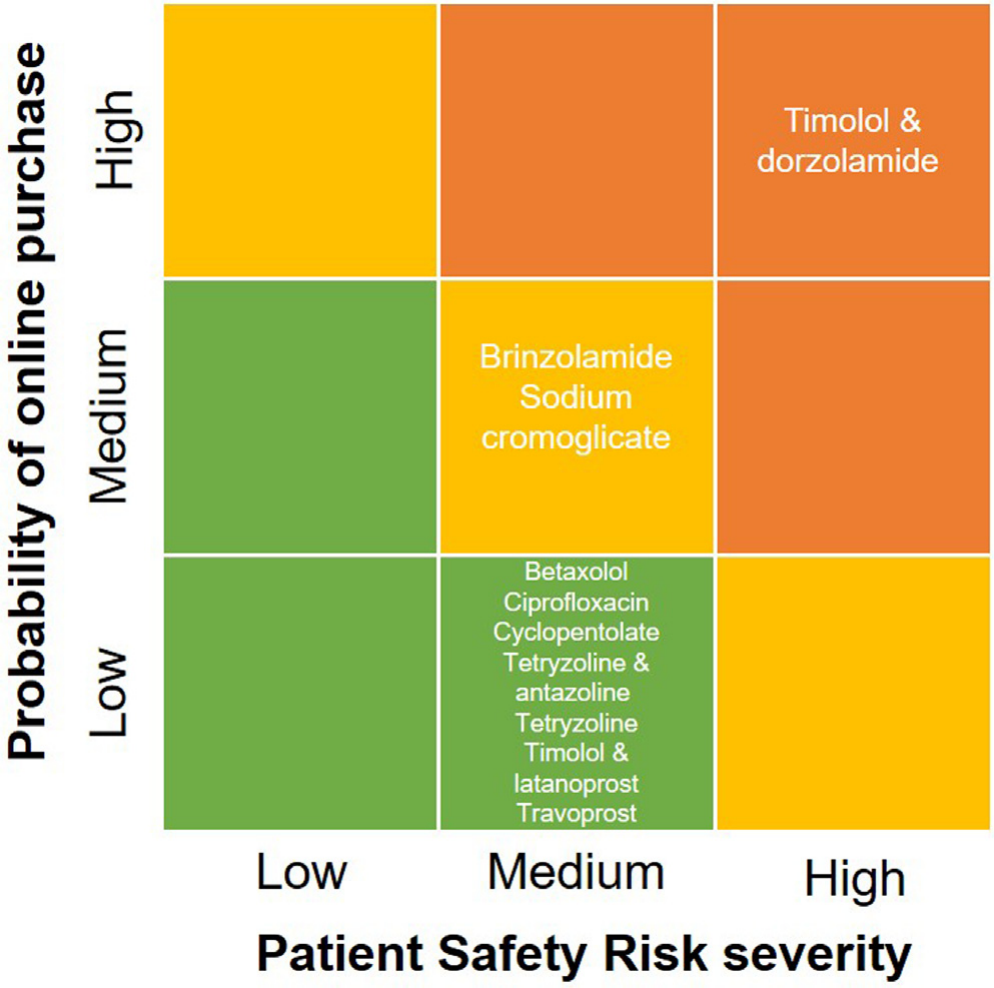
Risk assessment matrix for the selected eye drops.

Based on our complex risk assessment method, dorzolamide & timolol (S01ED51) eye drops were selected for test purchase with reaching the highest score in both categories, which is 10 points in the patient safety risk assessment and the highest probability score with 1 point. Three samples of timolol & dorzolamide eye drops were test purchased by the Authors in 2018 for detailed chemical and microbiological analysis. Fig. 1 illustrates the final risk assessment matrix consist of the patient safety risk score and the internet purchase probability score of the selected eye drops.

## 4 Discussion and conclusion

As it was mentioned in the introduction, 58.1% of the test purchases included some kind of preliminary selection parameters or criteria such as sales data or API with high ADR potential (European Alliance for Access to Safe Medicines (EAASM), 2008, Gelatti et al., 2013). Primarily researchers focused on products or APIs being popular in that days, systemic or fully explained motivations or methods were not discussed. Consequently there were many variables and the results originated from these studies were hard to compare without the same inclusion methods. Also four years ago our research group used the sales data and some motivations, but not in a standardized form. During the years we have had the motivation to develop a complex methodology called the risk based safety mapping of online pharmaceutical market, that was first introduced in 2017 in the International Journal of Clinical Pharmacy (Vida et al., 2017). In this article, we assumed there is a higher demand for somatropin products as being prescription-only medications and owning several unauthorized indications (e.g.: anti-aging) (Olshansky and Perls, 2008). The exact products were selected based on their national sales data. During the years we have not encountered any comprehensive method for preliminary risk assessment of medicines in the online pharmaceutical market. That is why we aimed to develop a complex and generally usable tool to measure patient safety risks and probability of the online medication purchase to select products for test purchases.

The main advantage of this newly developed method that it is a general framework with objective criteria regarding the pharmacological, technological, biopharmaceutical profile of the medication and the characteristics of the online internet market, so it can be used for other dosage forms and therapeutic categories as well. The authors think that the incorporation of the pharmacist perspective and that the tool can help to quantify patient safety risks in a rapid and objective way using readily available information sources is a great added value to the literature. However, the proposed framework is suitable for all drug classes, specific tailor-made modifications are required based on the type of research. Consequently, it is also cost-effective, since test procurement cannot be completed for all pharmaceuticals in the online pharmacy market. With an available low cost method the number of test purchases can multiple, accordingly this method may increase the overall safety of the online pharmaceutical market.

The methodology incorporates and emphasizes consumer perspective into the test procurement method when evaluating online pharmaceutical market. Furthermore, this complex methodology combines the characteristics of the products and the internet market. Hence, it can also be used in the legal internet sale of pharmaceuticals to identify products that should be counseled more thoroughly or medical follow up should be added when sold online by authorized pharmacies and pharmacists.

Additionally pharmacy owners or pharmacists responsible for this service of a community pharmacy can use this tool before starting an online pharmacy and screen the potential product portfolio (medicines and dietary supplements) to select products that can be sold safely via the internet. Beside the pharmacies, the pharmaceutical authorities will be able to use our tool to inspect the operation and patient safety of online vendors offering health care products. Also it can contribute to the pre-purchase vendor analysis studies and combined vendor, transport and product analysis to highlight the importance of microbiological analysis, as only two article included this perspective (Gelatti et al., 2013, Vida et al., 2017), however it should get more attention, especially during the COVID-19 pandemic.

Although, not just professionals but the patients and consumers can profit from this tool, as a simplified version can be used in patient-provider communication and promotion campaigns to prevent patient safety threats related to the online purchase of medications and other health care products, as previous surveys showed that several patients will purchase medications on the internet in the future (Fittler et al., 2018a)

Limitations of our research include that it illustrated by and focused on one dosage form, and locally to Hungary (commercially available products, shortage data, etc.). The selection process can be biased as it was based on an expert consensus and experience rather than exact national sales data.

The evaluation of the online pharmaceutical market has been in the focus of pharmacists and authorities in the last 20 years, which resulted in many regulations (e.g.: Falsified Medicine Directive and common EU logo), however their efficacy is debatable. That is why more and more unique techniques and methodologies are used to fight the illegal online vendors and protect patients and consumers. Beside these novel anti-counterfeiting approaches (“big data”, “infoveillance”, web crawling and deep learning models with artificial neural networks), further improvement of the traditional methods can also help the different actors in the combat against illicit online pharmacies. We think that our tool is applicable to prepare test purchase studies for academics and for authorities as well. Also the efficacy of joint health policy and forensic interventions (e.g.: PANGEA) can improve with a preliminary patient safety risk assessment.

Our future research directions include the publication of the quality and microbiological analysis of the ordered dorzolamide & timolol combination eye drops. Also we would like to continue the test purchases and the complex risk based safety mapping of online pharmaceutical market (see in the International Journal of Clinical Pharmacy Vida et al., 2017) and maybe expand and tailor our tool to the dietary supplement counterfeiting as well. Additionally we try to use this tool to the preliminary analysis and data source for the advanced computational methods (specified web crawlers) to detect and mitigate cybercriminal activity. We believe that in order to effectively prevent patient safety risks associated with the uncontrolled purchase of medication online, risk assessment based targeted interventions are required that can focus on products and active pharmaceutical ingredients with increased patient safety risks and active presence on the internet market.

## Declaration of Competing Interest

The authors declare that they have no known competing financial interests or personal relationships that could have appeared to influence the work reported in this paper.

## References

[b0005] Abbasi A., Zahedi F., Kaza S. (2012). Detecting fake medical web sites using recursive trust labeling. ACM Trans. Inf..

[b0010] Agarkhed M., O'Dell C., Hsieh M.C., Zhang J., Goldstein J., Srivastava A. (2018). Effect of surfactants on mechanical, thermal, and photostability of a monoclonal antibody. AAPS PharmSciTech.

[b0015] Agrahari V., Mandal A., Agrahari V., Trinh H.M., Joseph M., Ray A., Hadji H., Mitra R., Pal D., Mitra A.K. (2016). A comprehensive insight on ocular pharmacokinetics. Drug Deliv. Transl. Res..

[b0020] Alomar M.J. (2014). Factors affecting the development of adverse drug reactions (Review article). Saudi Pharm. J..

[b0025] American College of Clinical Pharmacy (ACCP), 2015. Pharmacotherapy Self-Assessment Program (PSAP) CNS/Pharmacy Practice. Adverse Drug Reactions. https://www.accp.com/docs/bookstore/psap/2015B2.SampleChapter.pdf (accessed 20.06.2020).

[b0030] American Society of Health System Pharmacists (1993). ASHP guidelines on preventing medication errors in hospitals. Am. J. Hosp. Pharm..

[b0035] Aronson J.K., Ferner R.E. (2017). Unlicensed and off-label uses of medicines: definitions and clarification of terminology. Br. J. Clin. Pharmacol..

[b0040] Ashames A., Bhandare R., Zain AlAbdin S., Alhalabi T., Jassem F. (2019). Public perception toward E-commerce of medicines and comparative pharmaceutical quality assessment study of two different products of furosemide tablets from community and illicit online pharmacies. J. Pharm. Bioallied Sci..

[b0045] Batchelor H.K., Marriott J.F. (2015). Formulations for children: problems and solutions. Br. J. Clin. Pharmacol..

[b0050] Baudouin C., Labbé A., Liang H., Pauly A., Brignole-Baudouin F. (2010). Preservatives in eyedrops: the good, the bad and the ugly. Prog. Retin. Eye Res..

[b0055] Blix H.S., Viktil K.K., Moger T.A., Reikvam A. (2010). Drugs with narrow therapeutic index as indicators in the risk management of hospitalised patients. Pharm. Pract. (Granada).

[b0060] Bowman C., Family H., Agius-Muscat H., Cordina M., Sutton J. (2019). Consumer internet purchasing of medicines using a population sample: A mixed methodology approach. Res. Social Adm. Pharm..

[b0065] Brudieu E., Duc D.L., Masella J.J., Croize J., Valence B., Meylan I., Mouillon M., Franco A., Calop J. (1999). Contamination bactérienne des collyres multi-doses: étude prospective au CHU de Grenoble [Bacterial contamination of multi-dose ocular solutions. A prospective study at the Grenoble Teaching Hospital]. Pathol. Biol. (Paris).

[b0075] Corazza O., Bersani F.S., Brunoro R., Valeriani G., Martinotti G., Schifano F. (2014). The diffusion of performance and image-enhancing drugs (PIEDs) on the internet: the abuse of the cognitive enhancer piracetam. Subst. Use Misuse.

[b0080] Davies N.M. (2000). Biopharmaceutical considerations in topical ocular drug delivery. Clin. Exp. Pharmacol. Physiol..

[b0085] Debnath B., Al-Mawsawi L.Q., Neamati N. (2010). Are we living in the end of the blockbuster drug era?. Drug News Perspect..

[b0090] Dedefo M.G., Mitike A.H., Angamo M.T. (2016). Incidence and determinants of medication errors and adverse drug events among hospitalized children in West Ethiopia. BMC Pediatr..

[b0095] European Alliance for Access to Safe Medicines (EAASM), 2008. The Counterfeiting Superhighway: The Growing Threat of Online Pharmacies. https://eaasm.eu/wp-content/uploads/455_EAASM_counterfeitingreport_0206081.pdf (accessed 6.15.20).

[b0100] European Association of Hospital Pharmacists (EAHP), 2018. EAHP’s 2018 Survey on Medicines Shortages to improve patient outcomes. https://www.eahp.eu/sites/default/files/report_medicines_shortages2018.pdf (accessed 27.06.2020).

[b0105] European Directorate for the Quality of Medicines (EDQM). 2016a. Resolution CM/Res(2016)1 on quality and safety assurance requirements for medicinal products prepared in pharmacies for the special needs of patients. https://www.edqm.eu/en/Quality-Safety-Standards-Resolutions-1588.html (accessed 6.15.20).

[b0110] European Directorate for the Quality of Medicines (EDQM), 2016b. Resolution CM/Res(2016)2 on good reconstitution practices in health care establishments for medicinal products for parenteral use. https://www.edqm.eu/en/Quality-Safety-Standards-Resolutions-1588.html (accessed 6.15.20).

[b0115] Eysenbach G. (1999). Online prescribing of sildanefil (Viagra) on the world wide web. J. Med. Internet Res..

[b0120] Farkouh A., Frigo P., Czejka M. (2016). Systemic side effects of eye drops: a pharmacokinetic perspective. Clin. Ophthalmol..

[b0125] Fittler A., Vida R.G., Káplár M., Botz L. (2018). Consumers turning to the internet pharmacy market: Cross-sectional study on the frequency and attitudes of Hungarian patients purchasing medications online. J. Med. Internet Res..

[b0130] Fittler A., Vida R.G., Rádics V., Botz L. (2018). A challenge for healthcare but just another opportunity for illegitimate online sellers: Dubious market of shortage oncology drugs. PLoS ONE.

[b0135] Fox E.R., McLaughlin M.M. (2018). ASHP guidelines on managing drug product shortages. Am. J. Health Syst. Pharm..

[b0140] Gabay M. (2015). Regulation of internet pharmacies: A continuing challenge. Hosp. Pharm..

[b0145] Gao X., Yang Q., Huang W., Chen T., Zuo C., Li X., Gao W., Xiao H. (2018). Evaluating eye drop instillation technique and its determinants in glaucoma patients. J. Ophthalmol..

[b0150] Gelatti U., Pedrazzani R., Marcantoni C., Mascaretti S., Repice C., Filippucci L., Zerbini I., Dal Grande M., Orizio G., Feretti D. (2013). 'You've got m@il: fluoxetine coming soon!': accessibility and quality of a prescription drug sold on the web. Int. J. Drug Policy.

[b0160] Hornof M., Toropainen E., Urtti A. (2005). Cell culture models of the ocular barriers. Eur. J. Pharm. Biopharm..

[b0165] Hughes, R.G., 2008. Patient Safety and Quality: An Evidence-Based Handbook for Nurses. Rockville (MD): Agency for Healthcare Research and Quality (US); Advances in Patient Safety. 21328752

[b0170] Institute for Safe Medication Practices, 2018. ISMP list of high-alert medications in acute care settings.

[b0175] International Pharmaceutical Federation (FIP), 2020. FIP and Patient Safety.

[b0180] Interpol. Operation Pangea, 2020. https://www.interpol.int/en/Crimes/Illicit-goods/Pharmaceutical-crime-operations (accessed 6.15.20).

[b0185] Iyer K., Dilipkumar N., Vasaya S., Pawar S., Diwan A. (2018). Comparison of drug related problems associated with use of narrow therapeutic index drugs and other drugs in hospitalized patients. J. Young Pharm..

[b0190] Jena A.B., Goldman D.P., Foster S.E., Califano J.A. (2011). Prescription medication abuse and illegitimate internet-based pharmacies. Ann. Intern. Med..

[b0195] Kadri R., Hegde S., Kudva A.A., Achar A. (2010). Over the counter ophthalmic drug misuse, are we aware?. Online J. Health Allied Scs.

[b0200] Kalyanam J., Katsuki T., Lanckriet R.G., Mackey T.K. (2017). Exploring trends of nonmedical use of prescription drugs and polydrug abuse in the Twittersphere using unsupervised machine learning. Addict. Behav..

[b0205] Kaushik K.S., Kapila K., Praharaj A.K. (2011). Shooting up: the interface of microbial infections and drug abuse. J. Med. Microbiol..

[b0210] Kyei S., France D., Asiedu K. (2019). Microbial contamination of multiple-use bottles of fluorescein ophthalmic solution. Clin. Exp. Optom..

[b0215] Labetoulle M., Frau E., Le Jeunne C. (2005). Systemic adverse effects of topical ocular treatments. Presse. Med..

[b0220] Lagan B.M., Dolk H., White B., Uges D.R., Sinclair M. (2014). Assessing the availability of the teratogenic drug isotretinoin outside the pregnancy prevention programme: a survey of e-pharmacies. Pharmacoepidemiol. Drug Saf..

[b0225] László P. (2007). Off-label alkalmazások (szakmai érvek, finanszírozási lehetôségek) [Off-label applications (professional rationales, financial possibilities)]. Neuropsychopharmacol. Hung..

[b0230] LegitScript, 2016. The Internet Pharmacy Market in 2016. Trends, challenges and opportunities. https://safemedsonline.org/wp-content/uploads/2016/01/The-Internet-Pharmacy-Market-in-2016.pdf (accessed 8.28.19).

[b0235] Li J., Xu Q., Shah N., Mackey T.K. (2019). A machine learning approach for the detection and characterization of illicit drug dealers on Instagram: Model evaluation study. J. Med. Internet Res..

[b0240] Liang B.A., Mackey T.K. (2009). Searching for safety: Addressing search engine, website, and provider accountability for illicit online drug sales. Am. J. Law Med..

[b0245] Liang B.A., Mackey T.K. (2012). Vaccine shortages and suspect online pharmacy sellers. Vaccine.

[b0250] Liang B.A., Mackey T.K. (2012). Online availability and safety of drugs in shortage: a descriptive study of internet vendor characteristics. J. Med. Internet Res..

[b0255] Mackey T.K., Liang B.A. (2012). Oncology and the internet: Regulatory failure and reform. J. Oncol. Pract..

[b0260] Mackey T.K., Aung P., Liang B.A. (2015). Illicit Internet availability of drugs subject to recall and patient safety consequences. Int. J. Clin. Pharm..

[b0265] Mackey T.K., Nayyar G. (2016). Digital danger: a review of the global public health, patient safety and cybersecurity threats posed by illicit online pharmacies. Br. Med. Bull..

[b0270] Mackey T.K., Nayyar G. (2017). A review of existing and emerging digital technologies to combat the global trade in fake medicines. Expert Opin. Drug Saf..

[b0275] Mackey T.K., Kalyanam J., Klugman J., Kuzmenko E., Gupta R. (2018). Solution to detect, classify, and report illicit online marketing and sales of controlled substances via Twitter: Using Machine learning and web forensics to combat digital opioid access. J. Med. Internet Res..

[b0280] Memmel L.M., Miller L., Gardner J. (2006). Over-the-internet availability of hormonal contraceptives regardless of risk factors. Contraception.

[b0285] Mitchell G.A., Dunnavan G. (1998). Illegal use of beta-adrenergic agonists in the United States. J. Anim. Sci..

[b0290] Morales D.R., Dreischulte T., Lipworth B.J., Donnan P.T., Jackson C., Guthrie B. (2016). Respiratory effect of beta-blocker eye drops in asthma: population-based study and meta-analysis of clinical trials. Br. J. Clin. Pharmacol..

[b0295] Mugoyela V., Mwambete K.D. (2010). Microbial contamination of nonsterile pharmaceuticals in public hospital settings. Ther. Clin. Risk Manag..

[b0305] National Institute of Pharmacy and Nutrition (NIPN), 2020a. Official authorized off-label licenses from the National Institute of Pharmacy and Nutrition Hungary. https://www.ogyei.gov.hu/egyeb_nyilvantartasok_listak (accessed 27.06.2020).

[b0310] National Institute of Pharmacy and Nutrition (NIPN), 2020b. Drug product database. https://ogyei.gov.hu/main_page (accessed 27.06.2020).

[b0315] Nayyar G.M.L., Breman J.G., Herrington J.E. (2015). The global pandemic of falsified medicines: laboratory and field innovations and policy perspectives. Am. J. Trop. Med. Hyg..

[b0320] Nayyar G.M.L., Breman J.G., Mackey T.K., Clark J.P., Hajjou M., Littrell M., Herrington J.E. (2019). Falsified and substandard drugs: Stopping the pandemic. Am. J. Trop. Med. Hyg..

[b0325] Nentwich M.M., Kollmann K.H., Meshack J., Ilako D.R., Schaller U.C. (2007). Microbial contamination of multi-use ophthalmic solutions in Kenya. Br. J. Ophthalmol..

[b0330] Neubert A., Dormann H., Prokosch H.U., Bürkle T., Rascher W., Sojer R., Brune K., Criegee-Rieck M. (2013). E-pharmacovigilance: development and implementation of a computable knowledge base to identify adverse drug reactions. Br. J. Clin. Pharmacol..

[b0335] Norbutas L. (2018). Offline constraints in online drug marketplaces: An exploratory analysis of a cryptomarket trade network. Int. J. Drug Policy.

[b0340] Olshansky S.J., Perls T.T. (2008). New developments in the illegal provision of growth hormone for “anti-aging” and bodybuilding. JAMA.

[b0345] Orizio G., Schulz P., Domenighini S., Caimi L., Rosati C., Rubinelli S., Gelatti U. (2009). Cyberdrugs: a cross-sectional study of online pharmacies characteristics. Eur. J. Public Health.

[b0350] Orizio G., Merla A., Schulz P.J., Gelatti U. (2011). Quality of online pharmacies and websites selling prescription drugs: a systematic review. J. Med. Internet Res..

[b0355] Petrova G., Stoimenova A., Dimitrova M., Kamusheva M., Petrova D., Georgiev O. (2017). Assessment of the expectancy, seriousness and severity of adverse drug reactions reported for chronic obstructive pulmonary disease therapy. SAGE Open Med..

[b0360] Piñero-López M.Á., Modamio P., Lastra C.F., Mariño E.L. (2016). Readability analysis of the package leaflets for biological medicines available on the internet between 2007 and 2013: An analytical longitudinal study. J. Med. Internet Res..

[b0365] Pullirsch D., Bellemare J., Hackl A., Trottier Y.L., Mayrhofer A., Schindl H., Taillon C., Gartner C., Hottowy B., Beck G., Gagnon J. (2014). Microbiological contamination in counterfeit and unapproved drugs. BMC Pharmacol. Toxicol..

[b0370] Raynor D.K., Veene P., Bryant D. (2014). The effectiveness of the summary of product characteristics (SmPC) and recommendations for improvement. Ther. Innov. Regul. Sci..

[b0375] Roth L., Adler M., Jain T., Bempong D. (2018). Monographs for medicines on WHO's model list of essential medicines. Bull. World Health Org..

[b0380] Saisyo A., Oie S., Kimura K., Sonoda K.H., Furukawa H. (2016). Microbial contamination of in-use ophthalmic preparations and its prevention. Bull. Yamaguchi Med. Sch..

[b0385] Saisyo A., Shimono R., Oie S., Kimura K., Furukawa H. (2017). The risk of microbial contamination in multiple-dose preservative-free ophthalmic preparations. Biol. Pharm. Bull..

[b0390] Sengupta P., Chatterjee B., Tekade R.K. (2018). Current regulatory requirements and practical approaches for stability analysis of pharmaceutical products: A comprehensive review. Int. J. Pharm..

[b0395] Smith G., Smith J.A., Brindley D.A. (2014). The falsified medicines directive: How to secure your supply chain. J. Generic Med..

[b0400] St George B.N., Emmanuel J.R., Middleton K.L. (2004). Overseas-based online pharmacies: a source of supply for illicit drug users?. Med. J. Aust..

[b0405] Sterling T., Irwin J.J. (2015). ZINC 15–ligand discovery for everyone. J. Chem. Inf. Model..

[b0410] Takahashi N., Tsuboi H., Yoshida N., Tanimoto T., Khan M.H., Kimura K. (2013). Investigation into the antinfluenza agent oseltamivir distributed via the internet in Japan. Ther. Innov. Regul. Sci..

[b0415] Teuchner B., Wagner J., Bechrakis N.E., Orth-Höller D., Nagl M. (2015). Microbial contamination of glaucoma eyedrops used by patients compared with ocular medications used in the hospital. Medicine (Baltimore).

[b0425] The National Association of Boards of Pharmacy, 2018. Internet Drug Outlet Identification Program Progress Report for State and Federal Regulators. https://nabp.pharmacy/wp-content/uploads/2018/02/Internet-Drug-Report-Feb-2018.pdf (accessed 8.28.19).

[b0430] Tsegaw A., Tsegaw A., Abula T., Assefa Y. (2017). Bacterial contamination of multi-dose eye drops at Ophthalmology Department, University of Gondar, Northwest Ethiopia. Middle East Afr. J. Ophthalmol..

[b0435] Uchegbu I.F., Florence A.T. (1996). Adverse drug events related to dosage forms and delivery systems. Drug Saf..

[b0440] US House of Representatives, Committee on Energy and Commerce, 2014. Hearing on counterfeit drugs. Memorandum. http://docs.house.gov/meetings/IF/IF02/20140227/101804/HHRG-113-IF02-20140227-SD002.pdf (accessed 27.06.2020.)

[b0450] Vaajanen A., Vapaatalo H. (2017). A single drop in the eye – effects on the whole body?. Open Ophthalmol. J..

[b0455] Veronin M.A., Lee E., Lewis E.N. (2007). “Insight” into drug quality: comparison of simvastatin tablets from the US and Canada obtained via the Internet. Ann. Pharmacother..

[b0465] Vida R.G., Fittler A., Mikulka I., Ábrahám E., Sándor V., Kilár F., Botz L. (2017). Availability and quality of illegitimate somatropin products obtained from the Internet. Int. J. Clin. Pharm..

[b0470] Vida R.G., Fittler A., Somogyi-Végh A., Poór M. (2019). Dietary quercetin supplements: Assessment of online product informations and quantitation of quercetin in the products by high-performance liquid chromatography. Phytother. Res..

[b0475] Wang F., Yu S., Liu K., Chen F.E., Song Z., Zhang X., Xu X., Sun X. (2013). Acute intraocular inflammation caused by endotoxin after intravitreal injection of counterfeit bevacizumab in Shanghai, China. Ophthalmology.

[b0480] World Health Organization (WHO), 2017. Definitions of Substandard and Falsified (SF) medical products. https://www.who.int/medicines/regulation/ssffc/definitions/en/ (accessed 17th July 2020).

[b0485] World Health Organization (WHO), 2020. Patient safety. https://www.who.int/patientsafety/about/en/ (accessed 20.06.2020).

[b0490] Zhou L., Rupa A.P. (2018). Categorization and association analysis of risk factors for adverse drug events. Eur. J. Clin. Pharmacol..

