# Supplementary material for: Developing a framework regarding a complex risk based methodology in the evaluation of hazards associated with medicinal products sourced via the internet
Source: Saudi Pharm J. 2020 Nov 6;28(12):1733–42. doi: 10.1016/j.jsps.2020.10.018 (PMC7783221; doi:10.1016/j.jsps.2020.10.018)
Supplement: Supplementary supplement 1 [file mmc1.docx]

**Table S1.: Literature overview of the internet test purchases published 1999-2020.**

| **No.** | **Date** | **Region** | **Therapeutic category or active ingredient** | **There was test purchase (Number of products purchased)** | **Preliminary method for the selection of model products** | **Reference** |
| --- | --- | --- | --- | --- | --- | --- |
| 1. | 1999 | USA | sildenafil citrate | Yes  (66 pills) | No | Eysenbach G. Online prescribing of sildanefil (Viagra) on the world wide web. J Med Internet Res. 1999;1(2):E10. doi: 10.2196/jmir.1.2.e10. http://www.jmir.org/1999/2/e10/ |
| 2. | 1999-2000 | USA | contraceptives | Yes  (10+15 items) | No | Miller L, Nielsen C. Internet availability of contraceptives. Obstet Gynecol. 2001 Jan;97(1):121–6.S0029-7844(00)01113-3 |
| 3. | 2003 | USA | fluoxetine hydrochloride, levothyroxine sodium, metformin hydrochloride, phenytoin sodium, warfarin sodium | Yes  (five samples of fluoxetine hydrochloride capsules (20 mg), two samples of levothyroxine sodium tablets (100 μg), four samples of metformin hydrochloride tablets (500 mg), three samples of phenytoin sodium capsules (100 mg), two samples of phenytoin sodium tablets (100 mg), and four samples of warfarin sodium tablets (5 mg)) | Five drug products were selected by the FDA Office of Compliance for inclusion in this study. | Westenberger BJ, Ellison CD, Fussner AS, Jenney S, Kolinski RE, Lipe TG, Lyon RC, Moore TW, Revelle LK, Smith AP, Spencer JA, Story KD, Toler DY, Wokovich AM, Buhse LF. Quality assessment of internet pharmaceutical products using traditional and non-traditional analytical techniques. Int J Pharm. 2005 Dec 8;306(1-2):56–70. doi: 10.1016/j.ijpharm.2005.08.027.S0378-5173(05)00606-X |
| 4. | 2004 | USA | simvastatin | Yes  (4 items) | No | Veronin MA, Youan BB. Medicine. Magic bullet gone astray: medications and the Internet. Science. 2004 Jul 23;305(5683):481. doi: 10.1126/science.1097355.305/5683/481 |
| 5. | 2004 | United States, Canada,  Argentina, Costa Rica, Fiji, India, Mexico, Pakistan, Philippines, Spain,  Thailand, Turkey. | isotretinoin, celecoxib, clozapine, lamivudine/zidovudine, indinavir, epoetin alpha, insulin, atorvastatin, oxycodone, acetaminophen/oxycodone, sildenafil, hydrocodone, sertralin | Yes  (68 samples) | No | US Government Accountability Office GAO. 2004. Jun 17, [2011-01-11]. Internet Pharmacies: Some Pose Safety Risks for Consumers http://www.gao.gov/products/GAO-04-820. |
| 6. | 2004-2005 | USA | levonorgestrel/ethinyl estradiol oral contraceptive, dropsirenon oral contraceptive ethinyl estradiol/norelgestromin patch | Yes  (3 Nordette® oral contraceptive, 3 Yasmin® oral contraceptive and 4 Orth-Evra® patch) | No | Memmel LM, Miller L, Gardner J. Over-the-internet availability of hormonal contraceptives regardless of risk factors. Contraception. 2006 Apr;73(4):372-5. Epub 2005 Dec 15. |
| 7. | 2005 | USA and Canada | Various products | No | The 44 brand-name medications most commonly purchased through the Internet from Canadian Internet pharmacies were included in the study. | Quon BS, Firszt R, Eisenberg MJ. A comparison of brand-name drug prices between Canadian-based Internet pharmacies and major U.S. drug chain pharmacies. |
| 8. | 2006 | USA | simvastatin | Yes  (four 20 mg tablets) | No | Veronin MA, Lee E, Lewis EN. "Insight" into drug quality: comparison of simvastatin tablets from the US and Canada obtained via the Internet. Ann Pharmacother. 2007 Jul;41(7):1111-5. Epub 2007 Jun 26. |
| 9. | 2006 | USA | oxycodone, hydrocodone, paracetamol and oxycodone, tramadol | Yes  (thirty 50 mg tablet tramadol) | Based on questionnaire with 1116 prescription drug abuser and drugs that are most commonly advertised online. | Cicero TJ, Shores CN, Paradis AG, Ellis MS. Source of drugs for prescription opioid analgesic abusers: a role for the Internet? Pain Med. 2008 Sep;9(6):718–23. doi: 10.1111/j.1526-4637.2007.00323.x.PME323 |
| 10. | 2006-2007 | USA | simvastatin, amlodipine, sildenafil | Yes  (18 generic simvastatin samples, 18 generic amlodipine samples, and 5 generic sildenafil samples) | commonly purchased pharmaceutical products | Veronin M. Packaging and labeling of pharmaceutical products obtained from the internet. J Med Internet Res. 2011 Feb 15;13(1):e22. doi: 10.2196/jmir.1441. |
| 11. | 2006-2007 | USA | simvastatin | Yes  (19 generic simvastatin 20 mg products purchased online) | No | Veronin MA, Nguyen NT. Comparison of simvastatin tablets from the US and international markets obtained via the Internet. Ann Pharmacother. 2008 May;42(5):613-20. doi: 10.1345/aph.1K560. Epub 2008 Apr 15. |
| 12. | 2007 | Canada | tadalafil, sibutramine, tramadol, diazepam, alprazolam;  Anatrium, Extra-time, Hoodia, Man XL | Yes  (five prescription drugs and four natural health products) | No | Gernburd P, Jadad AR. Will spam overwhelm our defenses? Evaluating offerings for drugs and natural health products. PLoS Med. 2007 Sep;4(9):e274. doi: 10.1371/journal.pmed.0040274. |
| 13. | 2008 | UK | tadalafil, vardenafil, sildenafil, finasteride, atorvastatin, clopidogrel, salmeterol/fluticasone, perindopril, telmisartan, tioptropium, olanzapin, venlafaxin, risperidon, donepezil, galantamin, lansoprazol, sibutramin, pramipexol | Yes  (36 items – 2 from each product) | This list of medicines was compiled by  identifying the top medicines in terms of US  sales. | European Alliance for Access to Safe Medicines EAASM. 2008. [2011-02-04]. The Counterfeiting Superhighway: The Growing Threat of Online Pharmacies https://eaasm.eu/public/downloads/6CJcZ/455_EAASM_counterfeiting+report_020608(1).pdf |
| 14. | 2008 | France | calcitriol, calcipotriol, tacalcitol, tazarotene, methotrexate, ciclosporine, acitretin, fumarate and methoxsalen, efaluzimab, alefacept, adalimumab, etanercept | No | We searched for all the treatments considered effective against psoriasis and suitable for outpatient use and available with prescription. | Mahé E, Saiag P, Aegerter P, Beauchet A. Shopping for psoriasis medications on the Internet. J Eur Acad Dermatol Venereol. 2009 Sep;23(9):1050-5. doi: 10.1111/j.1468-3083.2009.03248.x. Epub 2009 May 6. |
| 15. | 2008 | EU | legal highs | No | No | Hillebrand J, Olszewski D, Sedefov R. Legal highs on the Internet.  Subst Use Misuse. 2010 Feb;45(3):330-40. doi: 10.3109/10826080903443628. |
| 16. | 2008 | USA | Antibiotics  (azithromycin) | Yes  (six 500 mg azithromycin tablets) | The selected API was available without prescription in all of the identified internet pharmacies. | Mainous AG, Everett CJ, Post RE. et al. Availability of antibiotics for purchase without a prescription on the internet. Ann Fam Med 2009; 7: 431–5. |
| 17. | 2009 | USA | atorvastatin calcium, sildenafil citrate, celecoxib, esomeprazole magnesium, sertraline HCl | Yes  (A total of 152 drug orders were received, including 25 Celebrex®, 25 Lipitor®, 22 Nexium®, 50 Viagra®, 28 Zoloft® and two unknowns from two unidentifiable websites.) | The authors identified drugs most likely to be purchased by American consumers in several drug classes by cross-tabulating consumer self-reports with industry data, including lists of the most-popular online drug searches from licit website pharmacies and IMS's list of the top 10 products “most often prescribed” in the United States in 2007. | Bate R, Hess K. Assessing website pharmacy drug quality: safer than you think? PLoS One. 2010 Aug 13;5(8):e12199. doi: 10.1371/journal.pone.0012199. |
| 18. | 2009 | Japan | Diet-aid products containing Bukuryo (Poria sclerotium), Bakumondo (Ophiopogonis tuber), or Daio (rhubarb rhizome) | Yes  (15 oral items) | Using Japanese keywords for “personal import agent”, “slimming” and “obesity” (terms used in a list of “plant-origin materials used mainly as pharmaceutical medicine” created by the Ministry of Health, Labour and Welfare in Japan), we searched for diet-aid products that were described on Internet sites as containing Bukuryo (Poria, Poria sclerotium), Bakumondo (Ophiopogonis tuber), or Daio (rhubarb rhizome) on the Japanese Google search engine (www.google.co.jp).  Among the websites advertising such products, we selected those sellers that did not disclose their physical addresses and those that advertised the sale of falsified medicines (e.g., with unapproved dosages). | Yoshida N, Numano M, Nagasaka Y, Ueda K, Tsuboi H, Tanimoto T, Kimura K. Study on health hazards through medicines purchased on the Internet: a cross-sectional investigation of the quality of anti-obesity medicines containing crude drugs as active ingredients. BMC Complement Altern Med. 2015 Dec 4;15(1):430. doi: 10.1186/s12906-015-0955-2. |
| 19. | 2009 | Japan | Anti-obesity medicines | Yes  (82 samples) | We purchased one anti-obesity medicine that was listed first in one of the selected sites. In the subsequent selected websites, we purchased another brand or product of anti-obesity medicines, which was listed first. | Khan MH, Tanimoto T, Nakanishi Y, Yoshida N, Tsuboi H, Kimura K. Public health concerns for anti-obesity medicines imported for personal use through the internet: a cross-sectional study. BMJ Open. 2012 May 11;2(3). pii: e000854. doi: 10.1136/bmjopen-2012-000854. Print 2012. |
| 20. | 2009-2011 | South Africa, United States, China, Ethiopia, Thailand, Laos, Mexico, Nigeria and five Internet pharmacies | zidovudine, lamivudine, efavirenz, nevirapine, isoniazid and sulfamethoxazole/trimethoprim | Yes  (2027 tablets/capsules) | This list of medications was developed after reviewing medications that would be readily available at various geographical locations and after reviewing multiple international HIV treatment guidelines. | Wang T, Hoag SW, Eng ML, Polli J, Pandit NS. Quality of antiretroviral and opportunistic infection medications dispensed from developing countries and Internet pharmacies. J Clin Pharm Ther. 2015 Feb;40(1):68-75. doi: 10.1111/jcpt.12226. Epub 2014 Nov 10. |
| 21. | 2010 | USA | sildenafil citrate | Yes  (14 oral items containing 100 mg) | No | Veronin MA, Nutan MT, Dodla UK. Quantification of active pharmaceutical ingredient and impurities in sildenafil citrate obtained from the Internet. Ther Adv Drug Saf. 2014 Oct;5(5):180-9. doi: 10.1177/2042098614543091. |
| 22. | 2010 | Japan | oseltamivir phosphate | Yes  (34 samples of Tamiflu 75 mg and 9 of Antiflu 75 mg) | From 2009 to 2010, an influenza pandemic caused by influenza virus A (H1N1) occurred worldwide. | Takahashi N, Tsuboi H, Yoshida N, Tanimoto T, Khan MH, Kimura K. Investigation Into the Antinfluenza Agent Oseltamivir Distributed via the Internet in Japan. Ther Innov Regul Sci. 2013 Nov;47(6):699-705. doi: 10.1177/2168479013500966. |
| 23. | 2010 | USA | Dapoxetine HCl | Yes  (four 60 mg tablets) | No  (BetterSexTech.com site) | Dean J, Klep R, Aquilina JW. Counterfeit dapoxetine sold on the Internet contains undisclosed sildenafil. Int J Clin Pract. 2010 Aug;64(9):1319-22. doi: 10.1111/j.1742-1241.2010.02436.x. Epub 2010 May 20. |
| 24. | 2010-2011 | Italy | fluoxetine | Yes  (thirteen 20 mg pills) | Fluoxetine, a serotoninergic antidepressant, was chosen as target drug based on the following considerations. First of all, it belongs neither to the class of supplements nor to the so-called “life-style drugs” (e.g. Viagra®), which are particularly prone to counterfeiting (EAASM, 2008). The investigation could therefore focus on the specific portion of e-commerce represented by self-styled online pharmacies. Besides, being common, fluoxetine was not likely to be difficult to find on the Web. Moreover, it is a typical prescription drug, with specific indications and documented side effects including increased risk of suicide, risks associated with exposure in pregnancy, and withdrawal. | Gelatti U, Pedrazzani R, Marcantoni C, Mascaretti S, Repice C, Filippucci L, Zerbini I, Dal Grande M, Orizio G, Feretti D. 'You've got m@il: fluoxetine coming soon!': accessibility and quality of a prescription drug sold on the web. Int J Drug Policy. 2013 Sep;24(5):392-401. doi: 10.1016/j.drugpo.2013.01.006. Epub 2013 Feb 21. |
| 25. | 2011 | Japan | atorvastatin calcium | Yes  (six 10 mg tablets) | Here, Lipitor and its generic drugs containing atorvastatin calcium (ATC), used for the treatment of hyperlipidemia worldwide, were selected as a model prescription drug. | T Fukami, T Koide, H Hisada, et al. Pharmaceutical evaluation of atorvastatin calcium tablets available on the internet: a preliminary investigation of substandard medicines in Japan J Drug Deliv Sci Technol, 31 (2016), pp. 35-40 |
| 26. | 2011 | UK | isotretinoin | Yes  (eight 20 mg capsules) | Within funds available for the research, a purchasing request was submitted to the first eight illegitimate e-pharmacies (within the list of 50 surveyed), which reported selling Accutane in 20 mg capsules, did not require a prescription, did not require the consumer to register as a member and each had a different payment IP addresses. | Lagan BM, Dolk H, White B, Uges DR, Sinclair M. Assessing the availability of the teratogenic drug isotretinoin outside the pregnancy prevention programme: a survey of e-pharmacies. Pharmacoepidemiol Drug Saf. 2014 Apr;23(4):411-8. doi: 10.1002/pds.3565. Epub 2014 Feb 3. |
| 27. | 2011 | USA | sildenafil citrate | Yes  (twenty-two 100 mg tablets) | Pfizer Global Security monitored top search  results for the phrase “buy Viagra” on the leading  two Internet search engines from March 14–18,  2011. | Campbell N, Clark JP, Stecher VJ, Goldstein I. Internet-ordered viagra (sildenafil citrate) is rarely genuine. J Sex Med. 2012 Nov;9(11):2943-51. doi: 10.1111/j.1743-6109.2012.02877.x. Epub 2012 Aug 27. |
| 28. | 2011-2012 | USA | buprenorphine | No | No | Bachhuber MA, Cunningham CO. Availability of buprenorphine on the Internet for purchase without a prescription. Drug Alcohol Depend. 2013 Jun 1;130(1-3):238-40. doi: 10.1016/j.drugalcdep.2012.11.004. Epub 2012 Nov 30. |
| 29. | 2013 | USA | recalled drugs  (levomethadyl acetate hydrochloride, rofecoxib, pemoline, thioridazine, valdecoxib, ximelagatran, lumiracoxib, tegaserod maelate, rimonabant, dextropropoxyphene and propoxyphene napsylate, sibutramine hydrochloride, sitaxentan, aprotinin, efalizumab, gemtuzumab ozogamicin, drotrecogin alpha) | No | The first phase attempted to identify drugs subject to permanent recall or restricted access in two groups: (1) select large pharmaceutical markets; and (2) those listed as banned, withdrawn, severely restricted, or not approved by 24 countries as compiled by the United Nations. To accomplish this, we conducted a document and database review of DRA information sources and the 14th Issue of the UN Consolidated List of Products Whose Consumption and/or Sale Have Been Banned, Withdrawn, Severely Restricted or not Approved By Governments (“UN List”). | Mackey TK, Aung P, Liang BA. Illicit Internet availability of drugs subject to recall and patient safety consequences. Int J Clin Pharm. 2015 Dec;37(6):1076-85. doi: 10.1007/s11096-015-0154-8. Epub 2015 Jul 7. |
| 30. | 2013 | Japan | omeprazole | Yes  (28 oral items) | The Google Japan search engine was used to search for pharmacies offering omeprazole product. The searches were performed during the 19th August to 12th September 2013. The search terms used were ‘オメプラゾール and 個人輸入’ for Japanese sites and ‘Omeprazole and personal import’ for English sites. Among the hits, pharmacies offering omeprazole 20 mg tablets or capsules were selected. | Rahman MS, Yoshida N, Sugiura S, Tsuboi H, Keila T, Kiet HB, Zin T, Tanimoto T6 Kimura K. Quality of omeprazole purchased via the Internet and personally imported into Japan: comparison with products sampled in other Asian countries. Trop Med Int Health. 2018 Mar;23(3):263-269. doi: 10.1111/tmi.13028. Epub 2018 Jan 21. |
| 31. | 2014 | UK | diazepam, fluoxetine, simvastatin | No | The researchers selected these commonly used medicines (diazepam, fluoxetine and simvastatin), but did not complete any purchases. Diazepam is liable to abuse and impose risk of tolerance, dependence and withdrawal symptoms to consumers therefore it should be used in short-term under close monitoring. Fluoxetine has significant interactions with other medicines. Simvastatin has a number of important counselling points which should be relayed to the patient before use. | Alwon BM, Solomon G, Hussain F, Wright DJ. A detailed analysis of online pharmacy characteristics to inform safe usage by patients. Int J Clin Pharm. 2015 Feb;37(1):148-58. doi: 10.1007/s11096-014-0056-1. Epub 2015 Jan 7. |
| 32. | 2014 | Hungary | somatropin | Yes  (3 products: Omnitrope 10 mg/1,5 ml injection; 2 boxes of Genotropin 5,3 mg/ml powder and solvent for injection) | Products with significant national sales | Vida RG, Fittler A, Mikulka I, Ábrahám E, Sándor V, Kilár F, Botz L. Availability and quality of illegitimate somatropin products obtained from the Internet.  Int J Clin Pharm. 2017 Feb;39(1):78-87. doi: 10.1007/s11096-016-0398-y. Epub 2016 Nov 25.2014 |
| 33. | 2015 | USA | Seroquel 300 mg XR 100 pills (quetiapine), Abilify 20 mg 90 pills (aripiprazole), lamotrigine 200 mg 100 pills, lithium carbonate 300 mg 100 pills, and bupropion 150 mg SR 100 tablets | No | The drugs were selected based on recent prescribing trends for the treatment of bipolar disorder in the US. | Monteith S, Glenn T, Bauer R, Conell J, Bauer M. Availability of prescription drugs for bipolar disorder at online pharmacies. J Affect Disord. 2016 Mar 15;193:59-65. doi: 10.1016/j.jad.2015.12.043. Epub 2015 Dec 29. |
| 34. | 2015 | Japan | Vardenafil | Yes  (28) | No | Zhu S, Yoshida N, Kimura K, Matsushita R, Tsuboi H. Falsified vardenafil tablets available online. J Pharm Biomed Anal. 2020;177:112872. doi:10.1016/j.jpba.2019.112872 |
| 35. | 2015 | USA | Anabolic Androgenic Steroids | No | No | McBride JA, Carson CC 3rd, Coward RM. The Availability and Acquisition of Illicit Anabolic Androgenic Steroids and Testosterone Preparations on the Internet. Am J Mens Health. 2018 Sep;12(5):1352-1357. doi: 10.1177/1557988316648704. Epub 2016 May 11. |
| 36. | 2014-2016 | Hungary | oncology shortage drugs | No | Yes (based on national and international drug shortage lists) | Fittler A, Vida RG, Rádics V, Botz L. A challenge for healthcare but just another opportunity for illegitimate online sellers: Dubious market of shortage oncology drugs. PLoS One. 2018;13(8):e0203185. Published 2018 Aug 28. doi:10.1371/journal.pone.0203185 |
| 37. | 2016 | UK | Antibiotics | No | No | Boyd SE, Moore LSP, Gilchrist M, Costelloe C, Castro-Sánchez E, Franklin BD, Holmes AH. Obtaining antibiotics online from within the UK: a cross-sectional study. J Antimicrob Chemother. 2017 May 1;72(5):1521-1528. doi: 10.1093/jac/dkx003. |
| 38. | 2017 | UK | balcofen | No | No | Floyd CN, Wood DM, Dargan PI. Baclofen in gamma-hydroxybutyrate withdrawal: patterns of use and online availability. Eur J Clin Pharmacol. 2018 Mar;74(3):349-356. doi: 10.1007/s00228-017-2387-z. Epub 2017 Dec 3.2017 |
| 39. | 2017 | USA | mifepristone, misoprostol | Yes  (22 products - 20 mifepristone–misoprostol combination products and 2 that contained only misoprostol) | No | Murtagh C, Wells E, Raymond EG, Coeytaux F, Winikoff B. Exploring the feasibility of obtaining mifepristone and misoprostol from the internet. Contraception. 2018;97(4):287‐291. doi:10.1016/j.contraception.2017.09.016 |
| 40. | 2017. | Europe | New Psychoactive Substances (Cannabionoids) | Yes  (10 different products per country) | No | Tibor Markus Brunt, Amanda Marie Atkinson, Thomas Nefau, Magali Martinez, Emmanuel Lahaie, Artur Malzcewski, Martin Pazitny, Vendula Belackova, Simon D Brandt. Online Test Purchased New Psychoactive Substances in 5 Different European Countries: A Snapshot Study of Chemical Composition and Price. Int J Drug Policy 2017 Jun;44:105-114. doi: 10.1016/j.drugpo.2017.03.006. Epub 2017 May 1. DOI: 10.1016/j.drugpo.2017.03.006 |
| 41. | 2018. | United Arab Emirates | Furosemide | Yes  (1 product) | No | Akram Ashames, Richie Bhandare, Sham Zain AlAbdin, Tasnim Alhalabi, Farah Jassem. Public Perception toward E-commerce of Medicines and Comparative Pharmaceutical Quality Assessment Study of Two Different Products of Furosemide Tablets from Community and Illicit Online Pharmacies. J Pharm Bioallied Sci. 2019 Jul-Sep; 11(3): 284–291. doi: 10.4103/jpbs.JPBS_66_19 |
| 42. | 2018-2019 | Australia | modafinil | No | Yes (Psychonaut Web Mapping Research Group. Psychonaut web mapping project: alert on new recreational drugs on the web; building up a European‐wide web scan monitoring system, 2010.) | Dursun S, Dunn M, McKay FH. The availability and acquisition of modafinil on the internet.  Drug Alcohol Rev. 2019 Sep;38(6):699-702. doi: 10.1111/dar.12977. Epub 2019 Aug 16. |
| 43. | 2020. | Japan | tadalafil | Yes  (45 samples) | No | Sanada T, Yoshida N, Matsushita R, Kimura K, Tsuboi H. Falsified tadalafil tablets distributed in Japan via the internet. Forensic Sci Int. 2020;307:110143. doi:10.1016/j.forsciint.2020.110143 |
